# Left main coronary artery–Pulmonary trunk fistula incidentally detected during acute coronary infarction: Complementary roles of coronary angiography and CCTA

**DOI:** 10.1016/j.radcr.2026.09.068

**Published:** 2026-10-14

**Authors:** Hossam-Eddine Ejjebli, Soumia Boulouiz, Zakaria Bazid, Nabila Ismaili, Noha El Ouafi

**Affiliations:** a Faculty of Medicine and Pharmacy, Mohammed I^st^ University, Oujda, Morocco; b Department of Cardiology, Mohammed VI University Hospital/Mohammed I University, Oujda, Morocco; c Laboratory of Epidemiology, Clinical Research and Public Health, Faculty of Medicine and Pharmacy, Oujda, Morocco

**Keywords:** Coronary artery fistula, Left main coronary artery, Pulmonary artery, Coronary CT angiography, Congenital cardiac malformation, Case report

## Abstract

Coronary artery fistulas (CAFs) are rare vascular anomalies, and fistulas originating from the left main coronary artery (LMCA) are particularly uncommon. A 63-year-old woman with hypertension and obesity presented with acute anterior myocardial infarction and left ventricular systolic dysfunction. Coronary angiography incidentally identified a tortuous vascular tract arising from the proximal LMCA, but couldn’t define its drainage site. Coronary computed tomography angiography (CCTA) demonstrated a 3.9 cm fistulous tract with an origin and maximal diameter of 6 mm, coursing anteriorly and to the right of the pulmonary trunk before draining into it. No focal aneurysmal dilatation, mural thrombus, calcification, or secondary branches were identified. The pulmonary trunk measured 29 mm, the right-sided cardiac chambers were not dilated, and transthoracic echocardiography showed no noninvasive evidence of a large hemodynamically significant left-to-right shunt or right-sided volume overload. Percutaneous coronary intervention (PCI) was performed for the culprit left anterior descending lesion, after which the symptoms resolved. The clinical course favored the LAD lesion as the dominant cause of ischemia, although a contributory fistula-related steal phenomenon was not formally assessed. Following discussion among several cardiovascular experts, conservative clinical and echocardiographic surveillance was selected. This case highlights the complementary roles of coronary angiography and CCTA in defining fistula anatomy and supporting individualized management and follow-up planning.

## Introduction

Coronary artery fistulas (CAFs) are uncommon vascular anomalies that create an abnormal communication between a coronary artery and a cardiac chamber or great vessel, bypassing the myocardial capillary bed. Most are congenital, although acquired fistulas have been described after trauma, infection, cardiac surgery, or percutaneous coronary intervention. Their estimated prevalence is 0.002% in the general population, and they constitute 0.2–0.4% of congenital cardiac malformations. Fistulas arising from the left main coronary artery (LMCA) are exceptionally rare, accounting for about 0.7% of CAFs [1]. Most CAFs drain into low-pressure right-sided structures or the pulmonary artery rather than the left-sided chambers [2]. Their clinical significance is determined by anatomical configuration, fistula caliber, shunt magnitude, and associated coronary disease. Small fistulas are often asymptomatic, whereas larger lesions may cause dyspnea, angina related to coronary steal, arrhythmias, heart failure, pulmonary hypertension, thrombosis, or infective endocarditis. Management therefore ranges from clinical and imaging surveillance to transcatheter or surgical closure according to symptoms, hemodynamic impact, anatomy, and procedural feasibility [1]. Invasive coronary angiography remains central to fistula detection, especially during acute coronary syndrome evaluation, but its two-dimensional projections can incompletely define complex or tortuous tracts. Coronary computed tomography angiography (CCTA) complements angiography by providing high-resolution three-dimensional assessment of fistula origin, course, caliber, termination, and associated vascular abnormalities, which can help guide management [2]. This report describes an incidental proximal LMCA-to-pulmonary trunk fistula identified during acute coronary syndrome assessment and highlights the complementary diagnostic roles of angiography and CCTA.

## Case

We report the case of a 63-year-old woman with hypertension and obesity who was admitted to the cardiac intensive care unit with an acute anterior myocardial infarction. She had no known congenital heart disease and no previous cardiac intervention. On presentation, her blood pressure was 120/70 mmHg, heart rate was 100 beats/min, and oxygen saturation was 98% on room air. Cardiovascular examination revealed no audible continuous murmur. Electrocardiography demonstrated ST-segment elevation in the anterior leads (Fig. 1).

**Fig. 1:**
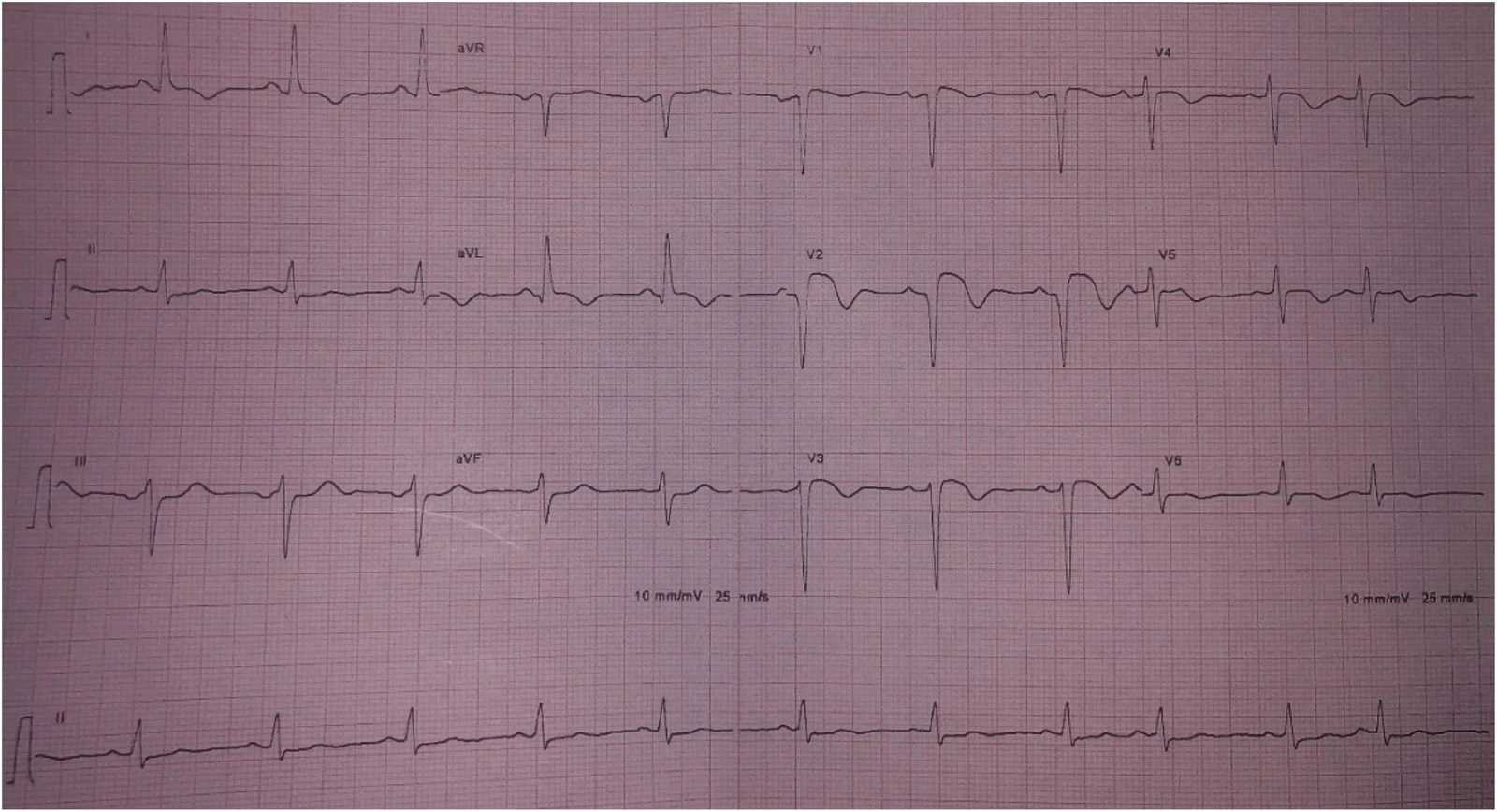
Twelve-lead electrocardiogram showing sinus rhythm, ST-segment elevation, and T-wave inversion in the anterior leads.

Transthoracic echocardiography demonstrated reduced left ventricular systolic function, with an ejection fraction of 38% and regional wall-motion abnormalities involving the anterior wall, without mechanical complication. The right-sided chambers were not dilated, and right ventricular systolic function was preserved. Tricuspid regurgitation (TR) was trivial, and the spectral Doppler envelope was adequate for measurement. Consequently, the recorded peak tricuspid regurgitant velocity (TR Vmax) of 1.0 m/s and the derived systolic pulmonary artery pressure (sPAP) of approximately 10 mmHg were interpreted cautiously because the weak signal could underestimate pulmonary pressure; this isolated value was not used as standalone evidence against hemodynamic significance. Color Doppler did not demonstrate a fistulous jet within the pulmonary trunk. However, the absence of a demonstrable jet on transthoracic echocardiography does not exclude the CCTA-confirmed fistula or a small-volume shunt (Fig. 2).

**Fig. 2:**
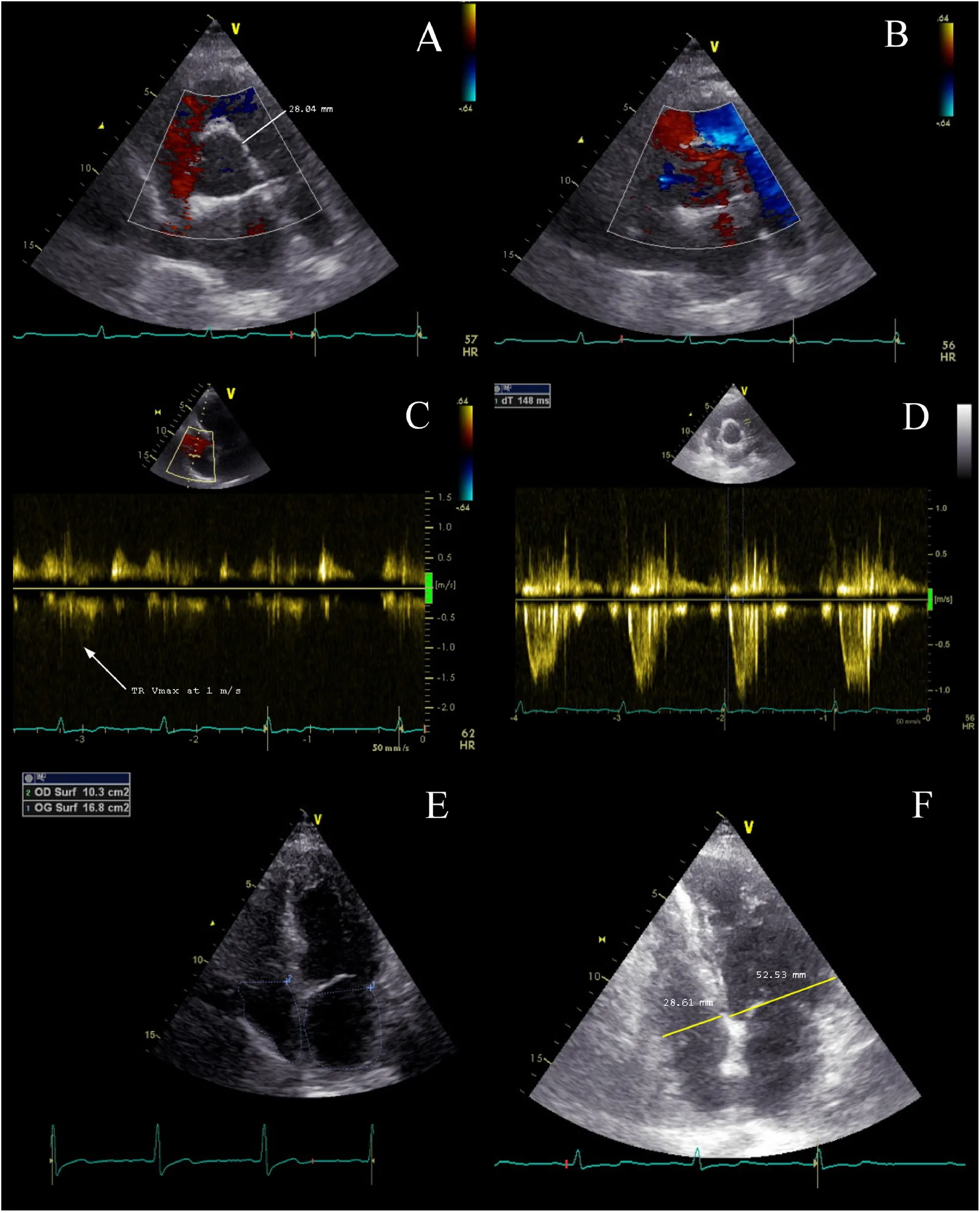
Transthoracic echocardiography assessment. (A–B) Parasternal short-axis views at end-diastole and end-systole showing no demonstrable fistulous jet on color Doppler; the pulmonary trunk measured 28 mm by echocardiography. (C) Peak tricuspid regurgitation velocity of 1.0 m/s, allowing estimation of an sPAP of 10 mmHg. (D) Doppler assessment showing no significant pulmonary regurgitation and a pulmonary artery acceleration time of 148 ms. (E–F) No right-sided chamber enlargement; the right atrial area was 10.3 cm², and the RV/LV ratio was < 0.6.

Coronary angiography showed an occlusion of the left anterior descending artery (LAD). A tortuous fistulous tract arising from the proximal LMCA was identified incidentally, but the drainage site could not be determined confidently on invasive angiography (Fig. 3).

**Fig. 3:**
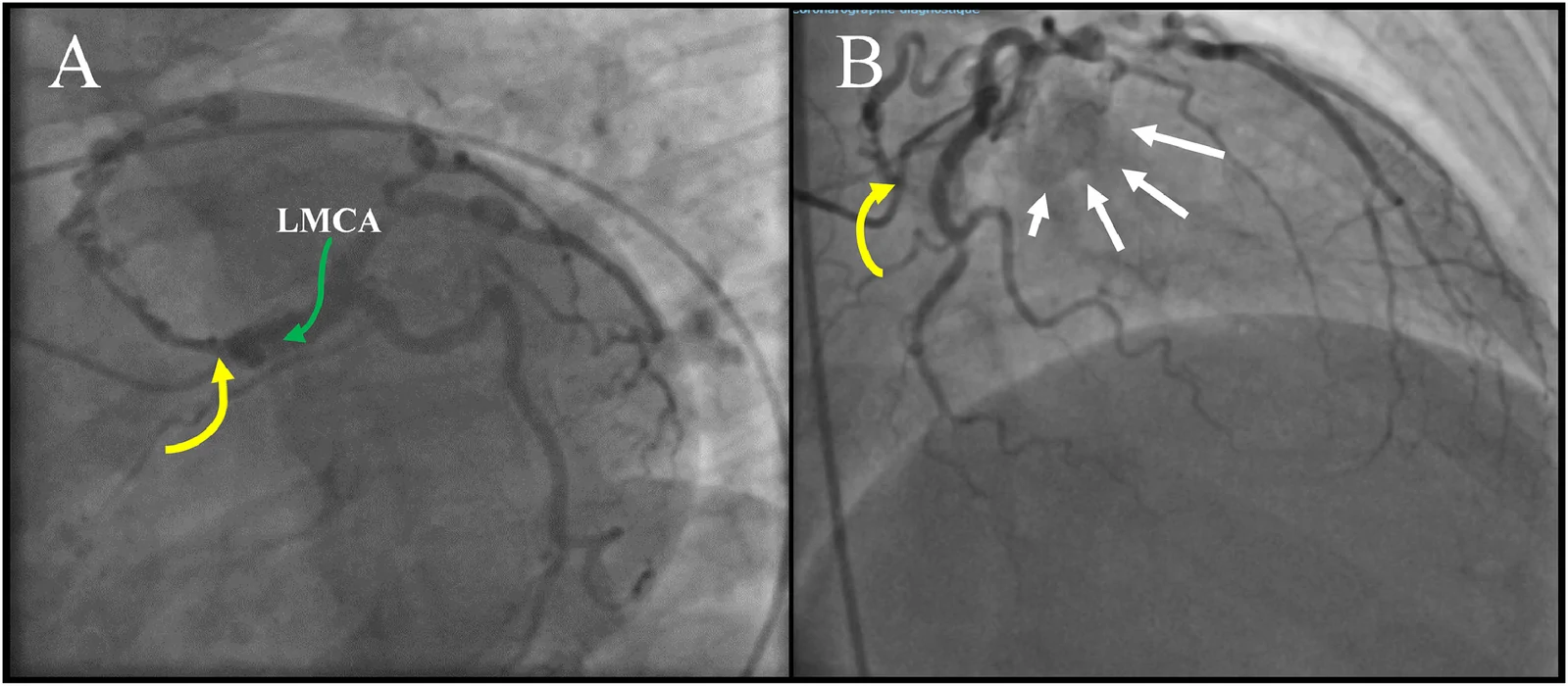
Coronary angiography showing (A) a tortuous fistulous tract (yellow arrow) arising from the proximal left main coronary artery (green arrow) and coursing toward the base of the heart, and (B) the proximal origin (yellow arrow) and progressive contrast opacification of the fistulous tract (white arrows) without clear angiographic identification of the drainage site.

Based on the concordance between the electrocardiographic findings and the coronary anatomy, the LAD was considered the culprit vessel. Percutaneous coronary intervention of the LAD was successfully performed, with restoration of TIMI grade 3 flow, and the patient’s chest pain resolved after revascularization.

CCTA was subsequently performed to define the fistula anatomy (Fig. 4). The tract originated from the proximal LMCA with an origin diameter of 6 mm, measured 3.9 cm in length, and had a maximal caliber of 6 mm. It coursed anteriorly and to the right of the pulmonary trunk before apparently terminating at its anterior aspect. No focal aneurysmal dilatation, mural thrombus, calcification, or identifiable secondary branches were identified. The main pulmonary artery measured 29 mm by CCTA, compared with 28 mm by transthoracic echocardiography; this minor difference was attributed to modality-specific acquisition planes and rounding rather than interval change. The right-sided chambers remained non-dilated.

**Fig. 4:**
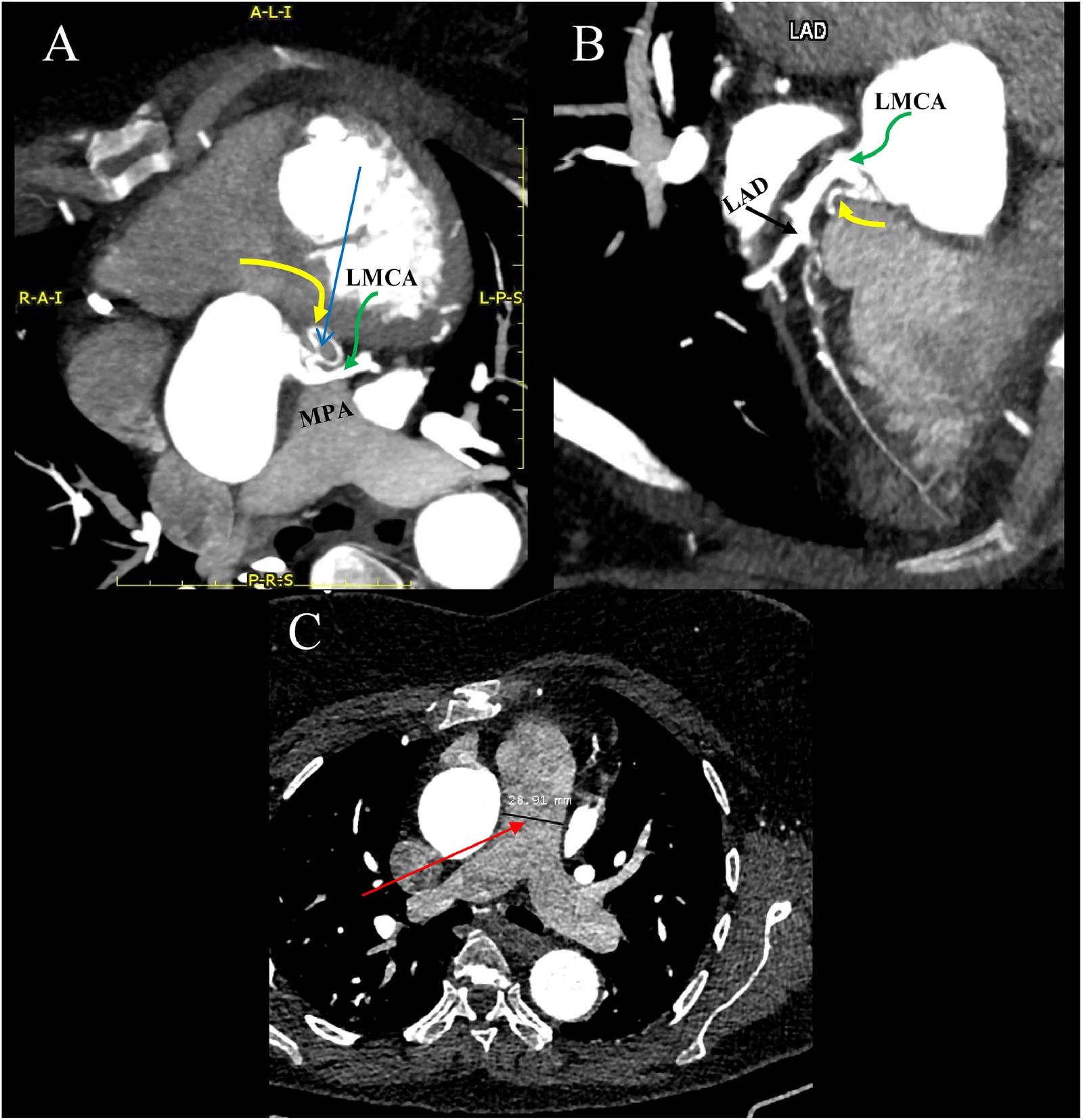
Coronary CT angiography assessment: (A) Double-oblique multiplanar reformatted image demonstrating the fistulous tract (yellow arrow) arising from the proximal left main coronary artery (green arrow) and coursing toward the pulmonary trunk before draining into it. (B) Axial image demonstrating the 6 mm origin of the fistulous tract (yellow arrow) from the proximal LMCA (green arrow) and its course toward the pulmonary trunk. The LAD is also visualized (black arrow). (C) Axial image at the level of the pulmonary arterial bifurcation showing a main pulmonary artery diameter of 29 mm (red arrow). No focal aneurysmal dilatation, mural thrombus, calcifications, or secondary branches were identified.

After revascularization, the patient remained clinically asymptomatic, without recurrent angina or dyspnea. This course favored the LAD occlusion as the dominant cause of ischemia. Because no functional ischemia test specifically assessing the fistula was performed, a contributory coronary steal phenomenon could not be completely excluded. Serial transthoracic echocardiography did not demonstrate a fistulous jet and showed no non-invasive imaging evidence of significant left-to-right shunting particularly no right-sided chamber enlargement or volume loading. Quantitative Qp/Qs assessment was not obtained; therefore, a small or moderate shunt could not be definitively excluded. Transesophageal echocardiography, cardiac magnetic resonance, and invasive hemodynamic assessment were deferred because the patient remained asymptomatic after LAD revascularization and had no indirect imaging evidence of significant right-sided volume loading. Following discussion among several cardiovascular experts, conservative clinical and echocardiographic surveillance was selected. At 6 and 12 months, the patient remained clinically stable and asymptomatic, and follow-up transthoracic echocardiography continued to show no dilated right-sided chambers without new indirect evidence of hemodynamic significance. Repeat CCTA was not performed; consequently, fistula caliber was not reassessed during follow-up. Repeat CCTA would be considered if new symptoms or echocardiographic abnormalities develop. Intervention would be reconsidered if surveillance demonstrates progressive fistula enlargement, right-sided chamber enlargement, quantitatively significant shunting, new or recurrent ischemia attributable to the fistula, or right ventricular dysfunction attributable to fistula-related loading.

## Discussion

Coronary artery fistulas (CAFs) are anatomically and clinically heterogeneous lesions whose consequences depend more on shunt burden and the receiving structure than on the site of origin alone. Although most fistulas arise from the right coronary artery or the left anterior descending artery, fistulas originating from the LMCA are distinctly uncommon, accounting for approximately 0.7% of cases [1]. Most fistulas drain into low-pressure right-sided structures or the pulmonary circulation rather than the left-sided chambers [3]. In the present case, the unusual feature was the combination of a proximal LMCA origin and a tortuous tract draining into the pulmonary trunk, whereas the drainage site itself remained within the expected distribution of coronary artery fistulas.

The hemodynamic significance of a CAF depends chiefly on the pressure gradient between the coronary artery and the drainage site, with the overall effect further influenced by fistula origin and maximal caliber, length, tortuosity, drainage site, aneurysmal change, mural thrombus. Management assessment should also consider right-sided chamber enlargement, directly quantified shunt burden, and functional evidence of coronary steal. In this patient, multimodality imaging revealed no right-sided chamber enlargement or other indirect evidence of a large hemodynamically significant left-to-right shunt. The main pulmonary artery measured 29 mm—slightly above the reported mean value in women (25.9 ± 3.0 mm) but below the published upper reference limit of 32.6 mm—and this isolated finding was insufficient to establish pathological pulmonary artery dilation [4]. Accordingly, further invasive hemodynamic quantification was deferred because the patient remained asymptomatic and showed no indirect evidence of right-sided volume overload. Fistula-specific functional ischemia testing was also deferred.

The presenting myocardial infarction was more plausibly attributable to the culprit LAD occlusion than to the fistula, because the electrocardiographic findings matched the coronary anatomy, PCI restored TIMI grade 3 flow, and symptoms resolved after revascularization. A contributory ischemic effect from coronary steal cannot be fully excluded [1], but the available clinical, angiographic, and echocardiographic findings did not strongly support that mechanism in this case.

Diagnostic imaging was central to anatomical characterization and management selection. Invasive coronary angiography has traditionally been the reference imaging technique and is frequently the modality by which a fistula is first identified [3], but its two-dimensional projections did not permit confident identification of the drainage site in this patient. CCTA resolved this uncertainty and documented the proximal LMCA origin, 6 mm origin and maximal caliber, 3.9 cm length, tortuous course, and apparent termination at the anterior aspect of the pulmonary trunk. These findings illustrate the value of multiplanar CCTA for defining coronary fistula anatomy and its relationship to adjacent cardiovascular structures [2,5].

Echocardiography provided complementary functional information by showing reduced left ventricular systolic function related to the infarction and non-dilated right-sided chambers without indirect evidence of a large shunt. When physiological significance remains uncertain, additional evaluation with transesophageal echocardiography, cardiac magnetic resonance, or invasive hemodynamic assessment can help quantify shunt burden more directly [6].

Reports of LMCA fistulas draining into the pulmonary circulation remain scarce. Gong et al. [7] described a case of a left main coronary artery-to-pulmonary artery fistula associated with aneurysmal dilatation of the fistulous tract. In contrast, no focal aneurysmal dilatation was identified in our patient, illustrating the morphological heterogeneity of this uncommon anatomical subtype.

The present case adds to that limited literature by demonstrating incidental detection during acute myocardial infarction evaluation and subsequent anatomical clarification with CCTA. The 6 mm caliber warranted continued surveillance for possible future aneurysmal enlargement, thrombosis endocarditis, or progressive steal; however, caliber alone was not considered an indication for closure in the absence of fistula-related symptoms, focal aneurysmal change, thrombus, right-sided volume loading, or indirect evidence of a large shunt. Following discussion among several cardiovascular experts, conservative management was selected. This approach was consistent with the contemporary guidelines principles favoring individualized assessment based on fistula-related symptoms, complications, shunt burden, anatomy, and expert review as the main indications for percutaneous or surgical closure [8]. This strategy was further supported by her asymptomatic clinical course after revascularization, follow-up at 6 and 12 months consisted of clinical assessment and transthoracic echocardiography only, repeat CCTA could be considered if symptoms or echocardiographic abnormalities emerge.

## Conclusion

Left main coronary artery fistulas are rare coronary anomalies that may be detected incidentally during evaluation for unrelated coronary disease. In this patient, invasive angiography raised suspicion for a proximal LMCA fistula, while CCTA was essential for defining its origin, tortuous course, caliber, length and drainage into the pulmonary trunk, thereby clarifying the anatomy beyond what angiography alone could establish. The available echocardiographic and imaging findings supported a conservative strategy, as the patient remained asymptomatic after LAD revascularization and showed no right-sided chamber enlargement without evidence of a large shunt on the available investigations. This case therefore illustrates how multimodality imaging, including transthoracic echocardiography and, when uncertainty persists, cardiac MRI or invasive hemodynamic assessment, helps define physiologic significance and guide individualized management ranging from surveillance to closure. In asymptomatic patients with stable clinical and radiologic findings, conservative follow-up is reasonable, but careful longitudinal surveillance remains important because these fistulas can evolve structurally over time, potentially warranting timely intervention.

## Data availability

Data sharing is not applicable to this article because no datasets were generated or analyzed during the current study.

## Ethical approval

Institutional ethics committee approval was not required for this single-patient case report in accordance with local policy.

## Author contributions

All authors contributed to the conception, drafting, critical revision, and final approval of the manuscript.

## Patient consent

Written informed consent was obtained from the patient for publication of the clinical details and any accompanying images.
